# Barriers and Facilitators to Engaging Mothers and Fathers in Family-Based Interventions: A Qualitative Systematic Review

**DOI:** 10.1007/s10578-022-01389-6

**Published:** 2022-06-28

**Authors:** Laura M. Jukes, Simona Di Folco, Lisa Kearney, Vilas Sawrikar

**Affiliations:** 1 School of Health in Social Science, The University of Edinburgh, Edinburgh, Scotland, UK; 2 National Health Service (NHS) Lothian, Edinburgh, Scotland, UK

**Keywords:** Family-based interventions, Parental engagement, Barriers, Facilitators, Qualitative studies

## Abstract

The current systematic review examined the similarities and differences between mothers’ and fathers’ reported barriers and facilitators to engaging in family-based interventions for child and adolescent behavioural problems (aged 2–17 years). Systematic searches of six electronic databases and grey literature alongside a two-way screening process identified twenty eligible qualitative studies from 2004 to 2019. A thematic meta-synthesis identified similarities in major themes of psychological, situational, knowledge/awareness, programme/intervention, co-parenting, practitioner, and beliefs/attitudes factors, alongside group experiences and stages of engagement. However, differences emerged in subthemes related to parental, treatment, and service delivery factors that included individual ideologies of parenting, parental roles, and treatment participation; the role of mothers in facilitating engagement; and individual preferences for treatment content and delivery. Overall, findings suggest that while mothers and fathers experience similar challenges to engagement, they can also experience distinct challenges which need to be addressed at the treatment outset to maximise engagement.

**Supplementary Information:**

The online version contains supplementary material available at 10.1007/s10578-022-01389-6.

Behavioural problems in children and adolescents (herein referred to as children or child) represent the most common reason for referral to mental health services [1]. Early-onset behaviour problems are also associated with lifetime trajectories of antisocial behaviour, substance abuse, and mood disorders, suggesting that early intervention for such problems is critical to preventing a life course of poor mental health [2]. Research suggests that the development of child behaviour problems is perhaps most significantly influenced by parenting whereby dysfunctional, coercive, and inconsistent parenting behaviours represent risk factors for adverse developmental outcomes [3]. Conversely, research shows that family-based interventions reliably reduce behaviour problems associated with negative parenting [4, 5]. These findings have generated interest in disseminating family-based interventions into the community to reduce the occurrence of behaviour problems in children [6].

Disseminating family-based interventions into the community requires engaging parents to seek help, attend, and participate in treatment [6]. On the contrary, research suggests that parents experience barriers to engaging in child mental health treatments [7]. Moreover, barriers to engaging in treatment may be experienced differently by mothers and fathers with evidence showing that fathers are less likely than mothers to attend and benefit from child treatment programmes [8, 9]. Research findings on barriers and facilitators to engaging parents in family-based interventions have been synthesised in previous reviews, leading to the formulation of putative strategies to maximise parental engagement [10–12]. However, much of the research reflects the views of mothers and does not report the views of fathers separately, which does not allow for the examination of potential differences or similarities between mothers and fathers in engaging in family-based interventions. To address this, the current study conducted a systematic review of qualitative studies to evaluate similarities and differences between mothers’ and fathers’ reported barriers and facilitators to engaging in family-based interventions. The term *parent*(*s*) for this review comprises biological mothers and fathers; non-biological relations such as stepparents, foster parents, and adoptive parents; and other biological or non-biological caregivers who serve in a parenting role. Thus, our paper utilises the terms *mother* and *father* to represent any adult of female or male sex who serve in a primary parenting role for children.

Despite significant treatment need among children, only a proportion of children and families seek help, engage, and participate in child mental health treatments. Epidemiological research examining engagement in child mental health treatment shows that only ten percent of children with mental health disorders seek professional help in mental health settings and only a third of families who begin treatment adequately attend sessions over time [13]. Meta-analytic reviews also show similar patterns of poor engagement in family-based interventions for child behaviour problems specifically, with research suggesting that half of identified families who would benefit from family-based interventions either do not enrol or drop out before completing treatment [14]. Important to the rationale for the current review are findings indicating that family-based interventions are mostly attended by mothers and that fathers are less likely to benefit from treatment [8, 15–17]. Effective engagement of both mothers and fathers is known to improve treatment outcomes, highlighting the need for strategies to address barriers to engaging mothers and fathers to ensure benefits from family-based interventions are realised [18].

The current review follows definitions of engagement in family-based interventions that recognise the need to include mothers, fathers, and the parenting system to maximise treatment outcomes. The Connect, Attend, Participate, Enact (CAPE) model [6] is one such framework that highlights the importance of considering the role of, and interaction between, mothers and fathers of a parenting system throughout various stages of treatment (i.e., recruitment, enrolment, retention, within-session involvement, between-session homework completion, implementation of learned parenting strategies). The CAPE model draws upon previous research showing that the participation of both mothers and fathers in family-based interventions is crucial to optimising child outcomes, such as reducing child aggression and disruptive behaviour, and improving child emotional regulation [19, 20]. Putative explanations for improved outcomes include consistency in the implementation of parenting strategies and supporting each other’s efforts which may, in turn, improve parenting satisfaction [16]. These findings reinforce the importance of engaging both mothers and fathers as significant agents of treatment in family-based interventions for reducing child behaviour problems.

Strategies addressing poor engagement in family-based interventions have developed from outcomes of research examining barriers and facilitators to parental engagement [21, 22]. In line with this, three previous systematic reviews have synthesised qualitative themes on parents’ barriers and facilitators to engagement in family-based interventions [10–12]. Common barriers included: fear of being judged/labelled as a bad parent; distrust or confidentiality concerns; competing demands (e.g., work commitments, childcare); lack of awareness of existing services; individual differences in group therapy (e.g., large sociodemographic differences between parents, language barriers, mixed parenting styles); inconvenient timing and location; lack of wider support network; feeling pressured to contribute to group discussion; programme content/goals not meeting parents’ expectations/needs; and parental conflict. Common facilitators included: a non-judgemental practitioner; acquiring parenting skills which encourage positive/desired child behaviour; content tailored to parents’ needs; peer support; incentives (e.g., refreshments, childcare); convenient timings and location, such as evening classes in the community; parenting strategies being ‘suggested’ rather than ‘dictated’; and home visits. Koerting et al. [11] review also found advertisement of family-based interventions via multiple platforms, direct recruitment (e.g., word-of-mouth between parents and between professionals and parents with established rapport), and multiple referral routes, as facilitators of parental engagement.

A major limitation of previous qualitative reviews is that fathers’ perceptions of barriers and facilitators to engagement in family-based interventions were not reported separately to mothers. For instance, most studies included in the reviews were based on mothers’ perceptions only or did not explicitly report differences between mothers and fathers (e.g., [23, 24]). Thus, engagement strategies formulated from these reviews could, paradoxically, side-line fathers as they do not include the perspective of fathers. Analysis of mothers’ and fathers’ reported barriers and facilitators may elucidate optimal methods for engaging parents by identifying differences that relate to the diversity of difficulties to engage in family-based interventions (e.g., [25, 26]). Such research by systematic review could inform recommendations for the provision of family-based interventions that meet the needs of both mothers and fathers to ensure effective treatment delivery in line with contemporary theories of parental engagement (e.g., CAPE model; [6]).

To this end, the current study consisted of a qualitative systematic review evaluating mothers’ and fathers’ reported barriers and facilitators to engaging in family-based interventions for child behavioural problems. This was done by analysing themes of barriers and facilitators of parental engagement for mothers and fathers separately via meta-synthesis and subsequently evaluating similarities and differences between mothers and fathers.

## Method

This review follows the Preferred Reporting Items for Systematic Reviews and Meta-Analyses (PRISMA) guidelines [27].

### Search Strategy

A systematic search of relevant electronic databases included PsychINFO, Embase, Ovid Medline, ProQuest, Scopus, and CINAHL. An additional hand-search of grey literature on Google Scholar alongside systematic searches on ProQuest were undertaken to identify evidence not published in commercial publications. Table 1 in Supplementary Information outlines the search strategy, including search terms, which was guided by the PICo framework; a methodological tool representing: (i) Population, (ii) Phenomena of Interest, and (iii) Context (e.g., Joanna Briggs Institute). Search terms were contained within titles, abstracts, keywords, and headings. No truncation indicators to make a singular word plural were used for the ‘Population’ component of PICo because each of its singular search terms (e.g., mother, father) were sufficient in generating relevant results. Five ‘Phenomena of Interest’ PICo components were used to function as ‘AND’—rather than ‘OR’—statements.

### Eligibility Criteria

#### Population

Studies were included if samples consisted of biological/non-biological parents/caregivers of a child between 2 and 17 years of age (thus, studies with only practitioners or children as participants were excluded). Where studies had children under 2 years of age in the sample, the study was included if the average child age in the sample was 2 years or greater. Studies not providing child ages had to define the family-based intervention as treating/targeting child behavioural/externalising difficulties or improving behavioural functioning in treatment. Female caregivers will hereafter be referred to as *mothers* while male caregivers will hereafter be referred to as *fathers*.

#### Phenomena of Interest

Studies were included if they reported mothers’ or fathers’ perceptions of barriers and/or facilitators to engagement in family-based interventions, as defined in the CAPE model [6]. Studies with both mothers and fathers as participants must have reported mothers’ perceptions separately to fathers’ perceptions. Studies consisting of mothers and fathers as participants but which did not report their barriers and facilitators to engagement separately were only included if there were 80% or more participants of the same sex and thus qualitative statements were considered the view of the majority sex (e.g., a study comprising 85% female participants would be assigned mothers’ perceptions overall). Family-based interventions designed to treat child behaviour problems or improve behavioural functioning and delivered face-to-face in any format (one-to-one, group, family, and multisystem) were included. No restriction was imposed on attendance status or the number of treatment sessions attended as our review sought to capture a broad range of experiences of parents and families who would benefit from treatment.

#### Context

Studies were included if they employed qualitative methods (focus groups, interviews, open-ended survey questions) to examine parents’ perceptions of barriers and facilitators to engagement in family-based interventions. Studies were peer-reviewed publications written in English. No date-range or country limits were imposed. All study designs were included.

### Study Selection

Search results from all six databases and a grey literature search were exported to, and deduplicated, on EndNote (a reference management programme). Two researchers (first and third author) independently performed a two-stage screening process on sourced studies to determine eligibility for inclusion in the current review. Stage one involved screening all titles and abstracts based on inclusion/exclusion criteria to identify potentially eligible papers for full-text screening in stage two. Ambiguous studies were also included for full-text review. Full-text screening in stage two was used to select final studies eligible for inclusion. There was complete agreement between the first and third author for inclusion criteria.

### Data Extraction

A data extraction tool was developed for this study based on methods used from previous qualitative systematic reviews [11, 12]. Data extraction was performed by the first author. Information extracted for analysis included intervention and sample characteristics, methods of data collection and analysis, and qualitative themes of barriers and facilitators to engagement as reported by mothers and fathers. In instances where barriers and facilitators were not explicit terms used, dislikes and preferences were proximal indicators of barriers and facilitators respectively (e.g., [23, 28]). Data was extracted from methods and results/findings sections of published studies.

### Quality Appraisal

Included studies were quality assessed after data extraction using the Critical Appraisal Skills Programme (CASP) for qualitative studies instrument. The overall quality of each study was determined using Butler et al. [29] CASP scoring system. The third author independently assessed quality for 25% of included studies. There was complete agreement between raters in relation to quality appraisal.

### Synthesis of Results

Meta-synthesis by the first author aggregated mothers’ and fathers’ reported barriers and facilitators to engagement in family-based interventions [30]. An integrative approach was adopted as themes within the primary studies were already specified, thus allowing deductive aggregation using thematic synthesis [31, 32]. Adoption of this approach was based on previous qualitative systematic reviews [10, 11]. Thomas and Harden’s [32] three stages of thematic synthesis guided the specific procedures used: (i) free line-by-line coding of study data, (ii) organisation of ‘free codes’ into related areas to develop descriptive themes, and (iii) development of analytical themes. Individual themes were translated from one study to the next (i.e., combining similar themes across qualitative studies) resulting in deductively aggregated themes across studies regarding barriers and facilitators of engagement in family-based interventions. Outcomes from aggregating themes for mothers and fathers via meta-synthesis are reported in Tables 4 and 5 in Supplementary Information (e.g., [11]). Tables 1 and 2 present shared and unique themes reported by mothers and fathers in relation to barriers and facilitators of engagement respectively. Similarities and differences in major themes (in bold) and subthemes (italicised) across mothers and fathers are identified based on shared and unique themes and reported in the results.

**Table 1:** Barriers to engagement in family-based interventions: Shared and unique themes reported by mothers and fathers

Major theme	Subtheme	Shared themes	Unique themes
Psychological factors	Stigma	Fear of being judged as a ‘bad parent’; shame/embarrassment of family problems	*Fathers*: Help-seeking considered as weak; fear of ridicule for attending; associating family-based interventions with Child Protection Services
	Distrust	Concerns with confidentiality	*Mothers*: Distrust of the practitioner’s affiliated system/organisation; social services investigations resulting from disclosures
	Parental mental health		*Mothers*: Experience of depression; ADHD; stress
	Attitudes/Beliefs		*Mothers*: Considering the intervention as non-beneficial; viewing the problem as within the child and not parenting; not ready/motivated/able to make changes to own behaviour; believing that missing a session means you cannot continue
Situational factors	Competing demands	Work commitments; busy schedule; childcare responsibilities	*Mothers*: Illnesses, caring for sick relatives; housework
	Practical	Transport difficulties; inconvenient timings	*Mothers*: Long waiting times; treatment affordability; lengthy homework tasks; child refusing to attend (i.e., multicomponent treatments)
	Demographic		*Mothers:* Single parent status; young parent; living in a disadvantaged community; having several children
Lack of knowledge/awareness*		Lack of knowledge and awareness of existing family-based interventions; unclear objectives and expectations of treatment	
Programme/Intervention experiences	Content	Treatment content not tailored to the needs of parents and children	*Mothers*: Overly clinical or spiritual language; not aligned to parents’ culture; strategies cannot be applied to several children. *Fathers*: Overly academic/lecture-based language; mother-focused; ‘too’ basic
	Poor orientation		*Mothers*: Poor orientation to the practitioner or service at the point of initial contact
	Negative past experiences		*Mothers*: Negative past experiences of mental health services; own experience of negative parenting
	Perceptions of inadequate treatment		*Mothers*: Perceived lack of family-based interventions for foster carers; perceived lack of improvement can lead to dropout
	Perceptions of family-based interventions		*Fathers:* Assumptions of users of family-based interventions; fewer opportunities for fathers to participate compared to mothers
Co-parenting*		Unsupportive co-parents; parental conflict	
Group therapy experiences	Group differences		*Mothers*: Differences in family demographics (e.g., age/education/income/marital status/socio-cultural); single parents not ‘fitting in’ with co-parents; differences in problem severity
	Fear/worries		*Mothers*: Fear of not ‘fitting’ in with the group; uncomfortable talking in front of a group; slipping into ‘old’ parenting styles without group support; low self-confidence in attending groups alone; overwhelmed by the quantity of parental involvement
Practitioner characteristics*			*Mothers*: Inability to manage group; poor parent-practitioner interactional style; inadequate understanding of child problems; inexperienced
Father involvement	Beliefs of fatherhood/masculinity		*Fathers*: Fathers not viewing themselves as primary caregivers; involvement in family-based interventions conflicts with traditional father/male ‘provider’ role; difficulties with expressing emotion
	Maternal gatekeeping		*Fathers:* Mothers controlling conversation; discouraging father participation; fathers becoming unwilling to share with mothers present

*Major theme without associated subthemes

**Table 2:** Facilitators of engagement in family-based interventions: Shared and unique themes reported by mothers and fathers

Major theme	Subtheme	Shared themes	Unique themes
Situational factors	Convenient location	Prefer local/community settings (e.g., school, community centres)	*Mothers*: Prefer home sessions; same location; non-threatening venue. *Fathers*: Prefer leaving home; attending varying locations; parking/transport access
	Convenient timings	Flexibility in therapy session timings; shorter therapy sessions	*Mothers*: Prefer fewer number of sessions overall; weekday evenings. *Fathers*: Prefer more sessions overall (e.g., to practice parenting strategies or have follow-ups); after-work hours; weekends
Knowledge/Awareness	Evidence-based intervention	Understanding that the intervention is supported by empirical evidence	
	Perceived intervention benefits	Perceiving the intervention to be beneficial; acknowledging improvements from participation	
Programme/Intervention experiences	Content	Tailored to the needs of parents and children	*Mothers*: Prefer practical strategies; structured; fun homework tasks. *Fathers*: Content on self-care; managing and normalising emotions; problem-solving challenging child behaviour; decision-making strategies; effective co-parenting; a focus on the father's role in child development
	Delivery	Easily accessible resources; comfortable environment; activity-based sessions	*Mothers*: Early provision of resources; resources provided in varying formats; one-to-one therapy. *Fathers*: In-person sessions combined with online resources; father-only groups; discussion groups
	Incentives	Reimbursement of travel expenses; refreshments; rewards/reinforcements for engagement	*Fathers*: Vouchers/gift-cards; childcare provision
	Additional support	Provide follow-ups	*Mothers*: Optional counselling for parents’ own mental health; refresher course; home visits; telephone session reminders; online forums
Co-parenting*		Participation of both mothers and fathers	*Mothers*: A focus on bonding/teamwork. *Fathers*: Mothers encouraging father participation
Group therapy experiences*		Peer support; learning from others; grouping parents with similar needs/experiences/struggles, trust/confidentiality	*Mothers*: Non-judgemental relationships between parents within a group
Practitioner characteristics*		Non-judgemental; inclusive of both parents; qualified/experienced/knowledgeable	*Mothers*: Manages group well; is a parent themselves; empowers parenting decisions; enquires about parent’s preferred interactional style. *Fathers*: Male practitioner for father-only groups; aware of child’s needs at outset; observes father-child interactions
Father involvement*			*Fathers:* Ideologies/beliefs regarding an actively and emotionally engaged father
Explicit engagement stages	Help-seeking/Enrolment:		*Mothers*: Feeling overwhelmed/helpless/desperate; family ‘crisis point’; recognised need for support; endeavouring to avoid child-welfare involvement; anticipating treatment benefits from attending. *Fathers*: Seeking to improve as a parent
	Treatment attendance/retention		*Mothers:* Rewards/reinforcements; incentives; motivation to complete treatment; improved co-parenting; perceiving that retention will aid parents’ mental illness; small attendance fee; experiencing/witnessing treatment benefits; culturally informed practice. *Fathers*: Seeking to improve as a parent
	Treatment in-session participation		*Mothers*: Improvements in co-parenting
	Enactment		*Mothers*: Offer of refresher courses/check-ins/follow-ups; co-parent/father/family support; incorporating learned strategies into daily routine
Advertisement/Recruitment	Various advertising formats	Use of multiple formats such as leaflets; word-of-mouth; hearing from previous attendees	*Mothers*: Access to information within their General Practice . *Fathers*: use of billboards; TV/radio/newspapers; endorsements by mothers, credible figures, or organisations
	Messages	Clear communication that orients parents to the intervention and participation requirements	*Fathers*: Father-friendly messages; father-relatable material; use of humour

*Major theme without associated subthemes

## Results

### Study Selection

**Fig. 1:**
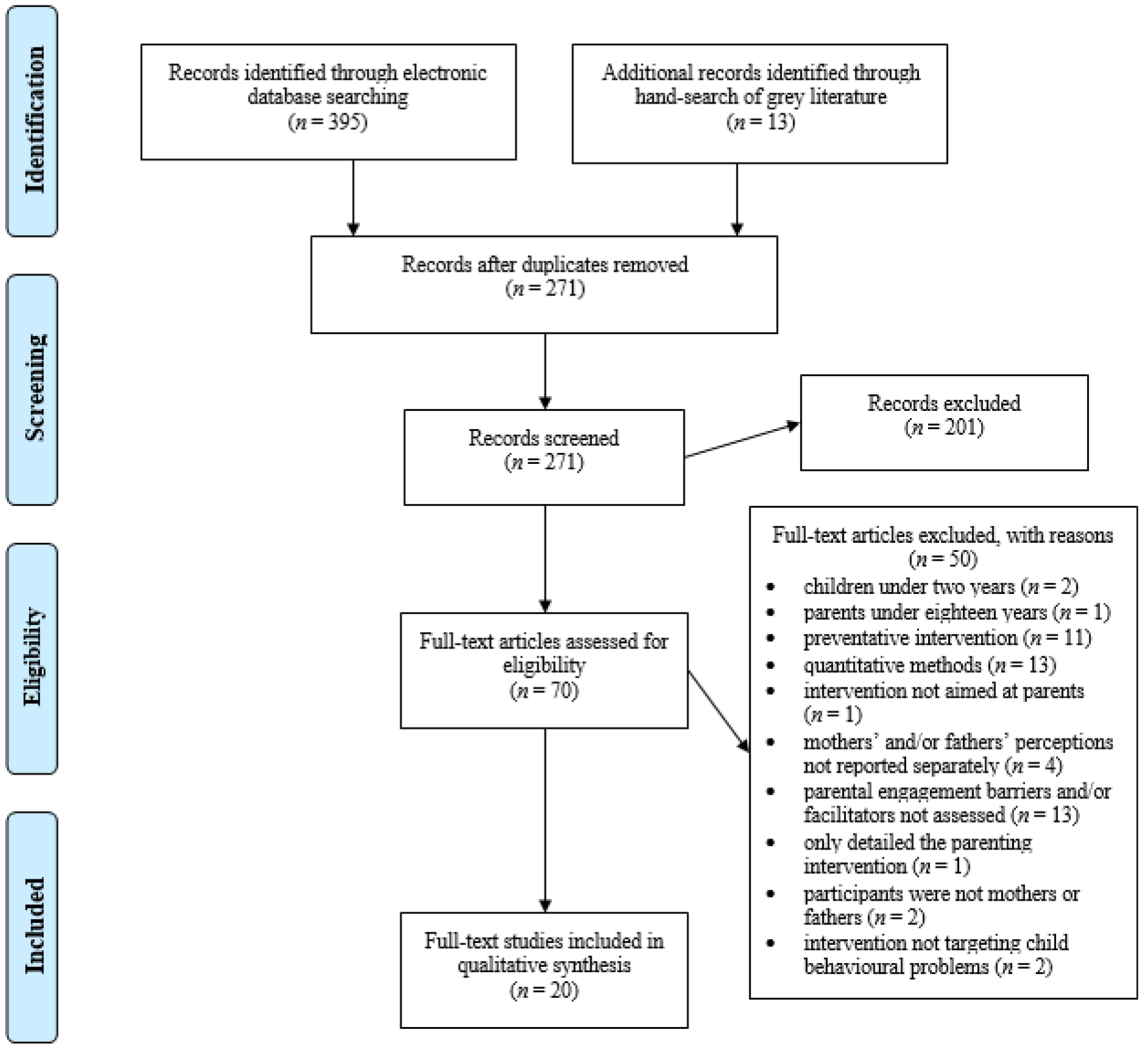
PRISMA diagram of selection process

Figure 1 outlines the PRISMA flowchart of the study selection process. The initial search strategy yielded 395 papers. A further 13 studies were identified through grey literature searches. Following the removal of duplicates, the total number of papers for screening was 271. Studies that were deemed to not satisfy the inclusion criteria were removed by title and abstract, resulting in 70 papers for full-text review. Of these 70 papers, 20 were eligible for inclusion based on study criteria.

### Characteristics of Included Studies

Characteristics of studies [26, 28, 33–50] included in this review are reported in Table 2 in Supplementary Information. Included studies were published between 2004 and 2019 and conducted in the UK (*n* = 7), USA (*n* = 6), Australia (*n* = 4), Ireland (*n* = 1), Sweden (*n* = 1), and New Zealand (*n* = 1). Barriers and facilitators of parental engagement were examined either in the context of specific family-based interventions (e.g., Triple P—Positive Parenting Programme) (*n* = 11), multi-family/system approaches (*n* = 4), father-only parenting programmes (*n* = 1), or parenting programmes for externalising problems or behavioural functioning (*n* = 4). Among the included studies, 12 studies reported mothers’ perceptions only; 6 studies reported fathers’ perceptions only; and 2 studies reported mothers’ and fathers’ perceptions on treatment engagement separately. There was a total of 345 parents across studies, with an interval number of 5 to 41 participants in a primary parenting role included in the 20 studies. Treatment attendance status was reported for 10 of the 20 included studies. Of the 170 parents included in the 10 studies: 7 (4.1%) had never attended a family-based intervention; 25 (14.7%) had completed the full treatment; 24 (14.1%) had not completed/dropped out of treatment; and the remaining 114 (67.1%) had completed one or more treatment sessions at the time of data collection.

There were 9 studies that reported the ethnic group of parents and 8 of these studies provided an ethnic group breakdown based on parents’ sex. Of these 8 studies, there were 88 mothers and 71 fathers whose separate ethnic groups were provided. Most fathers identified as African American (42.3%), followed by White English (15.5%), European (12.7%), Hispanic (9.9%), White (7%), Pacific Island (4.2%), Swedish (4.2%), Māori (2.8%), and Filipino (1.4%). Most mothers identified as Irish (35.2%), followed by White (26.1%), Black African American (13.6%), Hispanic/Latino (12.5%), Swedish (5.7%), Mixed Race (2.3%), Other ethnic group not specified (2.3%), Armenian (1.1%), and Syrian (1.1%). Of the 20 included studies, 10 reported ages of parents, which ranged from 18 to 60 years. Fourteen studies reported the age range of children in the study. Most interventions targeted nursery/primary school-aged children between 2 and 12 years of age (*n* = 8), followed by children and adolescents aged between birth and 2–16 years (*n* = 4), adolescents aged between 11 and 17 years (*n* = 1), and the early years of 3 months to 3 years of age (*n* = 1).

Data was collected through interviews (*n* = 13) and focus groups (*n* = 7). The most cited data analysis method was Thematic Analysis (*n* = 10), followed by Grounded Theory (*n* = 3), Content Analysis (*n* = 2), Interpretative Phenomenological Analysis (*n* = 2), Framework Analysis (*n* = 1), Malterud’s Method of Systematic Text Condensation (*n* = 1), and a modified inductive approach using multiple coders and analytical triangulation (*n* = 1).

### Quality Appraisal

Table 3 in Supplementary Information summarises outcomes from the quality assessment of included studies. The methodological rigour of included studies was assessed to be within the moderately high (*n* = 5) to high (*n* = 15) range. Key issues identified were that only 6 studies (30%) reported the relationship between researcher and participant (i.e., factors related to the researcher's own potential biases and influences throughout the recruitment and data collection processes), while 5 studies (25%) did not report ethics approval.

### Barriers to Engagement: Similarities and Differences in Themes Reported by Mothers and Fathers (Table 1)

#### Psychological Factors

Both mothers and fathers reported barriers related to psychological factors that included (i) *stigma* of being judged as a ‘bad parent’ and shame or embarrassment of having family problems. Fathers uniquely reported having concerns that help-seeking is a sign of weakness or not being able to cope, fear of ridicule for attending treatment, and concerns that family-based interventions are associated with Child Protection Services. Both mothers and fathers reported (ii) *distrust* in relation to confidentiality as a barrier. However, mothers uniquely reported distrust of the practitioner’s affiliated system and fear of disclosures leading to investigations from social services.

Further differences under psychological factors included mothers uniquely reporting (iii) *parental mental health,* referring to parents’ own experience of depression, Attention-Deficit/Hyperactivity Disorder (ADHD), or stress as impeding their engagement in treatment; and (iv) *attitudes/beliefs* regarding sessions not being beneficial; the problem being within the child and not parenting; not feeling ready, motivated, or able to make changes to own behaviour; and believing missing sessions meant you could not continue (e.g., because future content relates back to a missed session), as barriers to engagement.

#### Situational Factors

Both mothers and fathers reported situational barriers that included: (i) *competing demands* associated with work commitments, busy schedules, and childcare responsibilities. Mothers uniquely reported competing demands related to illnesses, caring for sick relatives, and housework. Both mothers and fathers reported (ii) *practical* barriers associated with transport difficulties and inconvenient timings. However, mothers also uniquely reported long waiting times for programme enrolment, lengthy homework tasks, their child refusing to attend (i.e., in multicomponent treatments), and treatment affordability (particularly amongst single mothers) as barriers to engagement.

Another difference within the major theme of situational factors was that mothers uniquely reported (iii) *demographic* factors of being a single or young parent, having several children, and living in a disadvantaged community where implementation of learned strategies is difficult in communities with high levels of antisocial behaviour.

#### Lack of Knowledge/Awareness

Both mothers and fathers reported lack of knowledge and awareness of family-based interventions as a barrier to engagement. Specifically, unclear treatment expectations and objectives were identified as barriers for both mothers and fathers within this major theme. No subthemes were identified for the major theme of lack of knowledge/awareness.

#### Programme/Intervention Experiences

Both mothers and fathers reported barriers related to (i) *content* not tailored to unique family needs. However, mothers uniquely reported overly clinical or spiritual language used in treatment and content not aligned with parents’ culture or applicable to several children within the family as barriers. Whereas fathers uniquely reported overly academic/lecture-based language used in treatment and content being mother-focused or ‘too basic’ as barriers to engagement.

Other differences in barriers within the major theme of programme/intervention experiences included mothers uniquely reporting (ii) *poor orientation* to the practitioner or service at the point of initial contact with the agency; (iii) *negative past experiences* of mental health services and parents’ own negative parenting history being triggered in sessions; and (iv) *perceptions of inadequate treatment* associated with perceived lack of family-based interventions for foster carers and increased likelihood of dropout when expected improvements did not occur quickly. By contrast, fathers uniquely reported (v) *perceptions of family-based interventions* are for more ‘legitimate’ users (e.g., mothers, problematic fathers, rich parents, or parents of children with serious behaviour problems), and perceiving fewer opportunities for fathers to participate in family-based interventions as barriers to engagement.

#### Co-parenting

Both mothers and fathers reported barriers associated with co-parenting issues in relation to unsupportive co-parents and parenting conflict. These barriers were noted to obstruct the implementation of learned strategies. No subthemes were identified for the major theme of co-parenting.

#### Group Therapy Experiences

Only mothers reported barriers related to group therapy experiences. Notably, mothers reported (i) *group differences* in family demographics (e.g., age, education, income, and socio-cultural), single parents not feeling like they ‘fit in’ with co-parents, and individual differences in child symptom severity as barriers to engagement. Mothers also reported (ii) *fears/worries* about not ‘fitting’ in with the group, feeling uncomfortable talking in front of a group, low self-confidence in attending groups alone, feeling overwhelmed by the quantity of parental engagement required, and worries about slipping into ‘old’ parenting strategies without group support.

#### Practitioner Characteristics

Only mothers reported barriers associated with practitioner characteristics, including inability to manage the group (e.g., poor time-management, not allowing everyone opportunities to speak), interactional style (e.g., language/cultural barriers), and inadequate understanding of child problems. No subthemes were identified for the major theme of practitioner characteristics.

#### Father Involvement

Only fathers reported barriers related to the major theme of father involvement. This included (i) *beliefs of fatherhood/masculinity* such as fathers not considering themselves as primary caregivers, perceiving active involvement in treatment conflicts with traditional ‘provider’ roles, and difficulties with expressing emotions. Another subtheme fathers reported was (ii) *maternal gatekeeping* referring to mothers controlling conversations in-session and discouraging father participation, leading to an unwillingness to share when the mother was present in sessions.

### Facilitators of Engagement: Similarities and Differences in Themes Reported by Mothers and Fathers (Table 2)

#### Practitioner Characteristics

For both mothers and fathers, facilitators included non-judgemental practitioners who were inclusive of both parents and were qualified/experienced/knowledgeable. However, mothers uniquely reported that engagement was facilitated by practitioners who empowered parents rather than dictated parenting decisions, enquired about the parent’s preferred interactional style, managed the group well, and were parents themselves. By contrast, fathers uniquely reported male practitioners for father-only groups, practitioners with awareness of their child’s needs, and practitioners who observed the child or father-child interactions as part of treatment as facilitators of engagement. No subthemes were identified for the major theme of practitioner characteristics.

#### Group Therapy Experiences

Both mothers and fathers reported facilitators that included positive group experiences of peer support, learning from others, trust/confidentiality, and grouping parents with similar needs/experiences (e.g., mothers reported that sharing experiences between parents reduced their ‘fear of judgement’ and helped them to feel less alone). Mothers uniquely reported egalitarian and non-judgemental relationships between parents as a facilitator of engagement. No subthemes were identified for the major theme of group therapy experiences.

#### Situational Factors

Both mothers and fathers reported facilitators related to situational factors that included (i) *convenient location* where programmes are delivered in local/community settings (e.g., school, community centres). Mothers uniquely reported programmes delivered at home or in the same location, and in non-threatening environments (e.g., bright learning spaces) as facilitators. Whereas fathers uniquely reported opportunities to leave home, programmes delivered in varying locations, and access to parking and transport as facilitators. Both mothers and fathers also reported (ii) *convenient timings* as a facilitator that included flexible/varying timings and shorter treatment session durations. Mothers uniquely reported sessions delivered on weekday evenings and fewer overall quantity of sessions as facilitators, while fathers sought sessions during after-work hours or at weekends and an increased overall quantity of sessions or follow-up consultation to practice parenting strategies.

#### Programme/Intervention Experiences

Both mothers and fathers reported programme/intervention experiences as facilitators that included (i) *content* tailored to the needs of parents and children. Mothers uniquely reported a preference for content that is structured with practical parenting strategies alongside fun homework activities. By contrast, fathers uniquely reported a preference for content emphasising the father’s role in child development, self-care, management/normalisation of emotions (e.g., learning how to show physical affection), decision-making strategies, problem-solving techniques for challenging child behaviour, and information on effective co-parenting. Both mothers and fathers reported facilitators related to (ii) *delivery* consisting of activity-based sessions with accessible resources delivered in comfortable environments. However, differences between mothers and fathers in this subtheme included mothers uniquely reporting a preference for accessing resources early in treatment via various formats and opportunities for individual therapy. By contrast, fathers uniquely reported sessions combining in-person therapy with online resources, discussion groups, and opportunities for father-only groups as facilitators.

Both mothers and fathers reported facilitators related to (iii) *incentives* that included reimbursement of travel expenses, refreshments, and rewards/reinforcements (e.g., fathers reported certificates of completion representing mastery). Fathers also uniquely reported a desire for vouchers/gift-cards and childcare provision to overcome situational barriers. Finally, both mothers and fathers reported (iv) *additional support* in the form of follow-up sessions (e.g., fathers perceived follow-up sessions to help troubleshoot specific issues) as facilitating engagement. Further, mothers uniquely reported refresher courses, home visits, telephone session reminders, online forums to aid engagement of parents who cannot attend in-person, and counselling to address parents’ own mental health, as facilitators.

#### Co-parenting

Both mothers and fathers reported the major theme of co-parenting as a facilitator of engagement. This included provisions for the participation of both mothers and fathers as a parenting team in treatment. Differences between mothers and fathers in this major theme included mothers uniquely reporting a desire to focus on bonding/teamwork within a parenting team and fathers uniquely reporting encouragement from mothers to participate. No subthemes were identified within the major theme of co-parenting.

#### Knowledge/Awareness

Both mothers and fathers reported facilitators related to knowledge/awareness that included: (i) increased knowledge of family-based interventions as an *evidence-based intervention*; and (ii) *perceived intervention benefits* in relation to anticipated improvements from participation. No differences between mothers and fathers emerged within this major theme and its associated subthemes.

#### Explicit Engagement Stages

Differences between mothers and fathers emerged in the context of specific engagement stages. For instance, mothers reported (i) *help-seeking/enrolment* to be facilitated by feeling overwhelmed, helpless, and desperate (particularly regarding improving their child’s behaviour), reaching family ‘crisis point’, recognising a need for support, endeavouring to avoid child-welfare involvement, and anticipating treatment benefits from attendance. By contrast, fathers reported *help-seeking/enrolment* to be facilitated by a motivation to want to improve as a parent. Furthermore, (ii) *treatment attendance/retention* was uniquely reported by mothers as facilitated by rewards/reinforcements, incentives, motivations to see the family-based intervention through, improved co-parenting, perceiving that retention will aid parents’ mental illness, experiencing/witnessing treatment benefits, a small attendance fee, and culturally informed practice. By contrast, *treatment attendance/retention* was uniquely reported by fathers as facilitated by a motivation to want to improve as a parent.

Other differences within the major theme of explicit engagement stages were that mothers uniquely reported (iii) *treatment in-session participation* supported by improvements in co-parenting, such as co-parent bonding and connecting with each other; and (iv) *enactment* of learned strategies enhanced by the availability of refresher courses/check-in’s/follow-up’s, co-parent/father/family support, and incorporating learned parenting strategies into daily routine.

#### Advertisement/Recruitment

Both mothers and fathers reported facilitators related to advertisement/recruitment that included: (i) use of *various advertising formats,* such as leaflets, word-of-mouth, and hearing experiences from previous attendees. Mothers uniquely reported preference for information at General Practices, while fathers uniquely reported preference for being informed about family-based interventions through mothers, billboards in neighbourhoods, TV/radio/newspapers, or credible figures and organisations. Another common facilitator for mothers and fathers included (ii) advertisements consisting of *messages* which communicate the requirements of treatment participation and orient to the intervention. However, fathers uniquely reported that engagement would be facilitated by *messages* that are father-friendly (e.g., not implying that fathers are doing a ‘bad job’), incorporate father-relatable material (e.g., images of fathers from different races/ethnicities), and use humour.

#### Father Involvement

Only fathers reported feeling more able to engage when they accepted the ideology of an actively and emotionally engaged father. No subthemes were identified for the major theme of father involvement.

## Discussion

The present review evaluated mothers’ and fathers’ reported barriers and facilitators to engaging in family-based interventions for the treatment of child behavioural problems. The aim was to identify individual barriers and facilitators to inform recommendations for enhancing the accessibility, fit, and effectiveness of family-based interventions for both mothers and fathers. Meta-synthesis of qualitative studies identified similarities and differences in themes reported by mothers and fathers. Key differences emerged in subthemes related to parental, treatment, and service delivery factors that are putatively important to improving parental engagement in family-based interventions.

The major themes identified in the current review suggest mothers and fathers generally report similar barriers and facilitators of engagement in family-based interventions. Consistent with previous reviews [10–12], for instance, our findings suggest that when accessing professional help, both mothers and fathers seek to feel heard and understood by practitioners and connected with parents who share similar parenting experiences, rather than have their parental role or family circumstance threatened by judgements. Importantly, mothers and fathers also reported preferences for interventions known to be effective, personalised to their needs, and accessible at a convenient location and time.

Outcomes of the current review also extend previous research by suggesting that mothers and fathers may also experience unique barriers and facilitators related to parental, treatment, and service delivery factors that can influence treatment engagement. In relation to parental factors, for instance, our review identified differences associated with perceived parenting roles (e.g., [51]). Specifically, mothers reported barriers due to competing demands regarding housework and caring for sick relatives, likely reflecting traditional ‘mother-carer’ roles in the family [52]. On the contrary, fathers reported perceiving help-seeking to be a sign of weakness or not coping as a barrier, which may have its roots in fathers’ perceptions of masculinity and related denial of weakness that reduces their willingness to participate in treatment [53]. Further, our review identified that fathers were less likely to engage if they did not view themselves as a primary caregiver or considered treatment participation to conflict with their ‘provider’ role. These results converge with previous reviews that fathers with traditional fatherhood/masculinity ideologies may struggle to embody caring roles in the family [52]. Relatedly, a facilitator of engagement for fathers was having attitudes of being an actively and emotionally engaged father. Taken together, the results support the need to assess fathers’ beliefs about their roles as a parent and promote a primary caregiving role for fathers in improving child outcomes in family-based interventions (e.g., [54, 55]).

Differences in parental factors were also reported in relation to help-seeking and treatment participation, highlighting differences in parents’ motivation to participate in treatment. For instance, fathers’ help-seeking/enrolment and treatment attendance/retention were supported by the motivation to improve as a parent. By contrast, mothers’ help-seeking/enrolment was facilitated by feelings of desperation for support with their child’s behavioural problems and anticipating such support to be beneficial. Furthermore, mothers reported that improvements in co-parenting and parents’ own mental health facilitated treatment attendance/retention. Taken together, these results suggest that practitioners should assess motivation for treatment for both mothers and fathers at the outset of referral to ensure they ‘Connect’ with parents in ways that optimise treatment outcomes [6, 16].

In relation to treatment-related factors, outcomes of the current review added support for individual mother and father directed content to improve engagement. Mothers reported preference for practical parenting strategies, structured material, and fun homework, while fathers reported preference for content covering self-care, emotions, problem-solving, co-parenting, and the role of fathers in child-rearing as important to facilitating engagement. Importantly, fathers reported ‘mother-focused’ content as a barrier in relation to intervention advertisement, design, and delivery, which supports proposed barriers by previous narrative reviews [16]. Indeed, fathers reported maternal gatekeeping, namely the encouragement or discouragement from mothers to participate in treatment, as a significant barrier to engaging in family-based interventions [56]. Thus, practitioners should elicit mothers' attitudes regarding father engagement to intervene with any maternal gatekeeping obstructing father participation [19]. There is also need for content designed to reflect the father’s role in child development, entailing underpinnings of the neurobiology of paternal behaviour (e.g., [57, 58]) and social influence (e.g., [59]), rather than content which assumes maternal and paternal roles are identical.

Finally, in relation to service delivery factors, outcomes of our review suggest that differences between mothers and fathers exist in how situational factors are experienced, thus service delivery should be tailored accordingly. For instance, fathers sought more flexibility with varying locations, including leaving home, and more opportunities to practice parenting strategies as identified by preference for more sessions and sessions available during after-work hours or at the weekend. On the other hand, mothers reported the same location, home environment, and fewer sessions overall on weekday evenings to facilitate engagement. These findings highlight the importance of assessing treatment preferences of both mothers and fathers, alongside supporting potential negotiations of session timings and location, including treatment delivery in community (non-clinical) settings [6].

Overall, findings of the current review highlight that mothers and fathers experience unique challenges to engaging in family-based interventions driven by gender-differentiated barriers and facilitators. Synthesis of the current results suggested that differences in barriers emerge in relation to parental, treatment, and service delivery factors that include perceptions of parental roles in the family, parenting, mother-focused content, and treatment participation, as well as the role of mothers in facilitating father engagement and preferences between mothers and fathers for treatment content and delivery. It should be noted that focusing on the differences here is not intended to overlook several similarities between mothers and fathers in reported barriers and facilitators of engagement. Our findings related to shared barriers and facilitators for mothers and fathers are consistent with existing theoretical frameworks dedicated to understanding predictors of parental engagement and how best to improve intervention fit and accessibility for parents in general [6, 60]. However, identifying differences between mothers and fathers enrich such frameworks with greater account of individual differences among parents. Our findings emphasise the need for content and delivery to be inclusive of both mothers and fathers in family-based interventions [6, 16].

## Strengths, Limitations, and Direction for Future Research

A key strength of this review related to the identification of unique challenges mothers and fathers experience in engaging in family-based interventions. However, further research is required to improve our understanding of barriers and facilitators for fathers since more studies reported on mothers (n = 14) compared to fathers (n = 8). It is important to acknowledge that this review included only two studies that reported the views of mothers and fathers separately. Therefore, reported differences in barriers and facilitators between mothers and fathers from this review may in part be accounted by different study characteristics. For instance, only studies conducted with mothers included participants with mental illness. Thus, the finding that mothers (vs fathers) consider engagement will improve their mental health, or that mothers (vs father) refer to parental mental health as a psychological obstacle to engagement, could be due to this difference in samples. The current review also included various family-based interventions delivered in different formats and targeting different developmental periods which increases the generalisability of findings. However, the current review provided limited information on specific stages of engagement. Taken together, we encourage future studies to use standardised methods (e.g., interview schedules) to elicit views on barriers and facilitators of engagement separately for mothers and fathers, especially in relation to the different stages of engagement (e.g., CAPE model).

Included studies were also limited in diversity of countries and ethnic group representation which limits generalisability of findings to diverse healthcare settings. It is important for future qualitative research to include ethnically diverse parents to ensure barriers and facilitators of ‘hard-to-reach’ parents are represented. Relatedly, the current review did not examine whether barriers and facilitators to engagement in family-based interventions may be experienced differently dependent on caregiver type (e.g., biological/non-biological parent, stepparent, foster/adoptive parent). To address this, future studies should assess barriers and facilitators to engaging in family-based interventions in relation to different caregiver types, separately for female and male caregivers.

In relation to methods, this review synthesised data deductively using meta-synthesis, meaning conclusions were drawn from existing themes. Therefore, the mere absence of a barrier or facilitator may not necessarily represent a ‘difference’. To address this, quantitative studies may help by testing specific hypothesis of relative importance regarding factors in understanding predictors of parental engagement. Future reviews may also wish to utilise framework synthesis whereby themes are synthesised in accordance with the parental engagement stages of the CAPE framework [6], thereby forming a theoretically coherent assessment of barriers and facilitators to engaging in family-based interventions.

## Summary

In summary, the current systematic review identified that mothers and fathers experience shared and unique barriers and facilitators to engaging in family-based interventions. Importantly, the findings suggest that differences emerge in relation to parental, treatment, and service delivery related factors which, if left unaddressed, may lead to parents feeling marginalised and unmotivated to participate in treatment which could significantly reduce the efficacy of treatment. The current results support practice that actively engages mothers, fathers, and parenting systems in treatment to address unique challenges in engaging parents. We encourage further mother-father comparative analyses to identify unique and shared barriers and facilitators to engaging in family-based interventions, using thematic analysis or framework analysis methods, to increase the coherence and translatability of our review’s findings into clinical recommendations.

### Supplementary Information

Below is the link to the electronic supplementary material.

Supplementary file1 (DOCX 113 KB)

## Data Availability

Data supporting the findings of this review are openly available within the studies included in this review’s analysis.
