# Supplementary material for: Barriers and Facilitators to Engaging Mothers and Fathers in Family-Based Interventions: A Qualitative Systematic Review
Source: Child Psychiatry Hum Dev. 2022 Jun 28;55(1):137–51. doi: 10.1007/s10578-022-01389-6 (PMC10796537; doi:10.1007/s10578-022-01389-6)
Supplement: Supplementary file 1 — Supplementary file1 (DOCX 113 KB) [file 10578_2022_1389_MOESM1_ESM.docx]

**Table 1 supplementary information.** Database search strategy

| 1. Population | Mother or Mum or Mom or Maternal or Father or Dad or Paternal or Caregiver or “Care-giver” or Coparent or “Co-parent” or Guardian |
| --- | --- |
| 2. Phenomena of Interest (a) | Barrier* or Obstruct* or Obstacle* or Hinder* or Facilitat* or Predict* or Promot* or Encourage* or Lever* or Motivat* or Drive* or Aid* or Help* or Limit* or Retention or Recruit* or Enrol* |
| 3. Phenomena of Interest (b) | Participation or Engagement or Involvement or Attendance or Inclusion |
| 4. Phenomena of Interest (c) | “Behav* management” or “Parent* Intervention” or “Parent* Program*” or “Parent* Training” or “Family Therapy” or “Family Intervention” |
| 5. Phenomena of Interest (d) | Child* or Adolescen* or Teen* or “Young Person” or Youth or Juvenile |
| 6. Phenomena of Interest (e) | “Mental Health” or External* or Conduct or Disrupt* |
| 7. Context | Qualitative or Theme* or Interview* or “Focus Group*” or Narrative* or Discourse* or Narration or Meaning* or Perspective* or Experience* or Belief* or Percept* or Perceive* or View* or Opinion* or Expectation* |
|  | 1 AND 2 AND 3 AND 4 AND 5 AND 6 AND 7 |

* represents a truncation indicator

**Table 2 supplementary information.** Characteristics of included studies

| Author (date) | Country | Family-based Intervention and its description | Age range of children whose parents are included in the study and/or which the intervention targets | Participant Characteristics | Reports Mothers’ and/or Fathers’ perceptions separately:  Yes/No | Outcome Measures | Qualitative Data Collection Method | Qualitative Data Analysis Method | Robustness |
| --- | --- | --- | --- | --- | --- | --- | --- | --- | --- |
| Attride-Stirling, Davis, Farrel, Groark and Day  (2004) | UK | The Lewisham Community Child and Family Service (LCCFS)  Description: Community-based intervention which aims to improve the self-worth, competencies and functioning of clients with mild to moderate difficulties, through collaborative work with children, their parents, and practitioners | 5 to 11 years | *Total number of parents*: 18 (11 completers of treatment; 7 non-completers of treatment)  *Sex of parents*: 16 females; 2 males *(note: subjective based on pseudonym given to parent)*  *Age range of parents*: Not provided  *Ethnic group of parents*: Not provided | No  All supporting quotes are reported as being from mothers; otherwise, mothers’ views and fathers’ views are not reported separately.  88.89% of participants are female  Assigned **mothers’ perceptions** | Experiences and expectations of LCCFS; reasons for completing or not completing LCCFS intervention; and accessibility and acceptability of the LCCFS to parents | Open-ended interview schedule | ATLAS/ti (text interpretation software package) and thematic analysis based on argumentation and grounded theory | High |
|  |  |  |  |  |  |  |  |  |  |

**Table 2** (continued)

| Author (date) | Country | Family-based Intervention and its description | Age range of children whose parents are included in the study and/or which the intervention targets | Participant Characteristics | Reports Mothers’ and/or Fathers’ perceptions separately:  Yes/No | Outcome Measures | Qualitative Data Collection Method | Qualitative Data Analysis Method | Robustness |
| --- | --- | --- | --- | --- | --- | --- | --- | --- | --- |
| Coates, Phelan, Heap and Howe  (2017) | Australia | Mental Health Positive Parenting Program (MHPPP); an adaptation of Triple P for parents with mental health illnesses  Description: 10-week parenting intervention to improve mental health outcomes, including reducing behavioural problems, for children of parents with a mental illness | 2 to 12 years | *Total number of parents*: 18  *Sex of parents*: Reported as being consistent with 86% females of the entire cohort  *Age range of parents*: Not provided  *Ethnic group of parents*: Not provided | No  All quotes/data are reported as being from mothers (*note: subjective based on pseudonym given to parent)*  Sex of parent reported as being consistent with 86% females of entire cohort  Assigned **mothers’ perceptions** | Experiences of MHPPP program, including components parents found helpful or unhelpful | Telephone interviews using open-ended questions | Thematic analysis | High |

**Table 2** (continued)

| Author (date) | Country | Family-based Intervention and its description | Age range of children whose parents are included in the study and/or which the intervention targets | Participant Characteristics | Reports Mothers’ and/or Fathers’ perceptions separately:  Yes/No | Outcome Measures | Qualitative Data Collection Method | Qualitative Data Analysis Method | Robustness |
| --- | --- | --- | --- | --- | --- | --- | --- | --- | --- |
| Dolan (2014) | UK | Five ‘dads only’ parenting programmes (delivered in the West Midlands, UK)  Description: Promotes sensitive and reflective parenting, and supports enhancing fathers’ knowledge of child behaviour/functioning relative to developmental trajectory | 11 months to 10 years  (average child age was 3 years old) | *Total number of parents*: 11 (parents had attended at least one of the five ‘dads only’ parenting programmes)  *Sex of parents*: All male  *Age range of parents*: 19 to 46 years  *Ethnic group of parents*: All White English | Yes  All participants are male/fathers  Assigned **fathers’ perceptions** | Motivations and challenges to participation; lack of knowledge regarding children and childcare; and changing thoughts and practices regarding fathering and fatherhood | Semi-structured interviews | Thematic analysis | High |

**Table 2** (continued)

| Author (date) | Country | Family-based Intervention and its description | Age range of children whose parents are included in the study and/or which the intervention targets | Participant Characteristics | Reports Mothers’ and/or Fathers’ perceptions separately:  Yes/No | Outcome Measures | Qualitative Data Collection Method | Qualitative Data Analysis Method | Robustness |
| --- | --- | --- | --- | --- | --- | --- | --- | --- | --- |
| Dorsey, Conover and Cox  (2014) | USA | McKay’s Engagement Intervention combined with Trauma-Focused Cognitive Behavioural Therapy (TF-CBT)  Description: Utilises strategies to overcome caregivers’ perceptual engagement barriers and targets trauma-specific child behavioural problems | Not provided; however, children referred to as ‘youth’ and the family-based intervention targets child/youth behavioural problems | *Total number of parents*: 7 (all are foster parents)  *Sex of parents*: *A*ll female  *Age range of parents*: Not provided  *Ethnic group of parents*: Not provided | Yes  All participants are female/mothers  Assigned **mothers’ perceptions** | Barriers and facilitators to participation and suggestions for further facilitating engagement | In-person interviews | Directed content analysis and conventional content analysis | High |

**Table 2** (continued)

| Author (date) | Country | Family-based Intervention and its description | Age range of children whose parents are included in the study and/or which the intervention targets | Participant Characteristics | Reports Mothers’ and/or Fathers’ perceptions separately:  Yes/No | Outcome Measures | Qualitative Data Collection Method | Qualitative Data Analysis Method | Robustness |
| --- | --- | --- | --- | --- | --- | --- | --- | --- | --- |
| Frank, Keown, Dittman and Sanders (2015) | New Zealand | Parenting programs  Description: Parenting interventions based on social learning principles designed to treat child behavioural problems | 2 to 9 years | *Total number of parents*: 15  *Sex of parents*: All male  *Age range of parents*: Not provided  *Ethnic group of parents*: 9 (60%) European; 3 (20%) Pacific Island; 2 (13%) Māori; and 1 (7%) Filipino | Yes  All participants are male/fathers  Assigned **fathers’ perceptions** | Fathers’ preferences for program content and delivery, and fathers’ suggestions about increasing attendance and involvement in parenting programs | Focus groups | Inductive approach for analysis of qualitative evaluation data. Meaningful statements extracted to create categories and further defined into sub-themes | High |

**Table 2** (continued)

| Author (date) | Country | Family-based Intervention and its description | Age range of children whose parents are included in the study and/or which the intervention targets | Participant Characteristics | Reports Mothers’ and/or Fathers’ perceptions separately:  Yes/No | Outcome Measures | Qualitative Data Collection Method | Qualitative Data Analysis Method | Robustness |
| --- | --- | --- | --- | --- | --- | --- | --- | --- | --- |
| Friars and Mellor (2009) | Australia | Six parent training programmes  Description: Behaviour management programmes for parents of children diagnosed with Oppositional Defiant Disorder, Conduct Disorder, or Attention-Deficit/Hyperactivity Disorder (ADHD) | 4 to 8 years | *Total number of parents*: 9 (all parents had dropped out of treatment)  *Sex of parents*: 8 females; 1 male  *Age range of parents*: Not provided  *Ethnic group of parents*: Not provided | No  All supporting quotes are explicitly reported as being from mothers; otherwise, mothers’ views and the one father’s views are not reported separately.  88.9% of participants are female  Assigned **mothers’ perceptions** | Reasons why parents drop out of child behaviour management programmes | Interviews | Responses were grouped into themes | Moderate |

**Table 2** (continued)

| Author (date) | Country | Family-based Intervention and its description | Age range of children whose parents are included in the study and/or which the intervention targets | Participant Characteristics | Reports Mothers’ and/or Fathers’ perceptions separately:  Yes/No | Outcome Measures | Qualitative Data Collection Method | Qualitative Data Analysis Method | Robustness |
| --- | --- | --- | --- | --- | --- | --- | --- | --- | --- |
| Furlong and McGilloway  (2012) | Ireland | Incredible Years BASIC Preschool/Early School Years Parent Training Program (IYP)  Description: Parenting intervention supporting improvements in parent-child interactions and child behavioural outcomes | Children whose parents attended had mean age of 4 years and 9 months  Intervention targets children aged 3 to 7 years | *Total number of parents*: 33 (25 attended ten or more of the fourteen treatment sessions; and 8 dropped out after attending less than five treatment sessions)  *Sex of parents*: 31 females; 2 males  *Age range of parents*: Not provided; however, parents had a mean age of 34 years  *Ethnic group of parents*: All Irish | No  All supporting quotes are explicitly reported as being from mothers; otherwise, mothers’ views and fathers’ views are not reported separately.  93.3% of participants are female  Assigned **mothers’ perceptions** | Aspects of the program which were valued by parents and produced positive changes; challenges parents encountered in learning new skills; and experiences of parents who dropped out | One-to-one semi-structured interviews | Constructivist grounded theory in order to elicit and organise themes | High |

**Table 2** (continued)

| Author (date) | Country | Family-based Intervention and its description | Age range of children whose parents are included in the study and/or which the intervention targets | Participant Characteristics | Reports Mothers’ and/or Fathers’ perceptions separately:  Yes/No | Outcome Measures | Qualitative Data Collection Method | Qualitative Data Analysis Method | Robustness |
| --- | --- | --- | --- | --- | --- | --- | --- | --- | --- |
| Fox, Bibi, Millar and Holland(2017) | UK | Multisystemic Therapy (MST)  Description: An intensive family and community-based intervention facilitating positive change within the system of adolescents engaging in antisocial behaviour, such as via strengthening parenting strategies to improve family and adolescent functioning | Not provided; however, intervention defined as treating adolescent behaviour problems | *Total number of parents*: 7  *Sex of parents*: 6 females; 1 male  *Age range of parents*: 2 in their forties; 4 in their fifties; and 1 in their sixties  *Ethnic group of parents*: 1 (14%) Rwandan; 1 (14%) Jamaican; 1 (14%) Israeli; 2 (29%) Ghanaian; 1 (14%) Chinese; and 1 (14%) Irish | No  All supporting quotes are reported as being from mothers (*note: subjective based on pseudonym given to parent*); otherwise, mothers’ views and fathers’ views are not reported separately  85.7% of participants are female  Assigned **mothers’ perceptions** | Experiences of MST and factors which facilitated or hindered parental engagement | Interviews | Charmaz’s social constructivist version of grounded theory | Moderate |

**Table 2** (continued)

| Author (date) | Country | Family-based Intervention and its description | Age range of children whose parents are included in the study and/or which the intervention targets | Participant Characteristics | Reports Mothers’ and/or Fathers’ perceptions separately:  Yes/No | Outcome Measures | Qualitative Data Collection Method | Qualitative Data Analysis Method | Robustness |
| --- | --- | --- | --- | --- | --- | --- | --- | --- | --- |
| Gopalan, Fuss and Wisdom  (2015) | USA | Multiple Family Group (MFG) service delivery model  Description: Research informed intervention for child welfare-involved families to treat child behavioural problems, including disruptive behaviour disorders | Not provided; however, intervention defined as treating child behavioural problems | *Total number of parents*: 25 (4 attended 0-25% of treatment; 5 attended 26-50% of treatment; 8 attended 51-75% of treatment; and 8 attended 76-100% of treatment)  *Sex of parents*: All female  *Age range of parents*: 26-57 years  *Ethnic group of parents*: 12 Black African American; 11 Hispanic/Latino; and 2 Other (not reported) | Yes  All participants are female/mothers  Assigned  **mothers’ perceptions** | Factors influencing decision to enrol and remain in the intervention; previous experiences with child mental health services and community resources; and suggestions for improving service/intervention delivery | Semi-structured interviews | Grounded theory | High |

**Table 2** (continued)

| Author (date) | Country | Family-based Intervention and its description | Age range of children whose parents are included in the study and/or which the intervention targets | Participant Characteristics | Reports Mothers’ and/or Fathers’ perceptions separately:  Yes/No | Outcome Measures | Qualitative Data Collection Method | Qualitative Data Analysis Method | Robustness |
| --- | --- | --- | --- | --- | --- | --- | --- | --- | --- |
| Huntington and Vetere  (2015) | UK | Parenting programmes  Description: Promotes positive parenting practices to improve parent-child relationships and child behaviour | Target children of parents included in the study were 3 months to 3 years  Intervention targets children under 5 years, and aims to improve child behaviour | *Total number of parents*: 12 (i.e., 6 coparenting teams who had participated in a parenting programme)  *Sex of parents*: 6 females; 6 males  *Age range of parents*: Not provided  *Ethnic group of parents*: Not provided | Yes  Assigned **mothers’ and fathers’ separate perceptions** | Mothers’ and fathers’ perspectives on coparenting practices and suggestions for adapting interventions to meet the needs of parents / facilitate parental engagement | Semi-structured interviews with coparenting teams | Interpretative Phenomenological Analysis (IPA) | Moderate |

**Table 2** (continued)

| Author (date) | Country | Family-based Intervention and its description | Age range of children whose parents are included in the study and/or which the intervention targets | Participant Characteristics | Reports Mothers’ and/or Fathers’ perceptions separately:  Yes/No | Outcome Measures | Qualitative Data Collection Method | Qualitative Data Analysis Method | Robustness |
| --- | --- | --- | --- | --- | --- | --- | --- | --- | --- |
| Kerwin, Giorgio, Steinman and Rosenwasser  (2014) | USA | Behavioural Parenting Training (BPT)  Description: An evidence-based parenting intervention for child welfare-involved families to treat parent and child behaviour problems | 1 to 10 years  Intervention targets child behavioural problems | *Total number of parents*: 23  *Sex of parents*: All female  *Age range of parents*: Not provided  *Ethnic group of parents*: Not provided | Yes  All participants are female/mothers  Assigned **mothers’ perceptions** | Perspectives on BPT; barriers hindering participation in BPT; and facilitators to participation in BPT | Focus groups | Identified themes from transcriptions | Moderate |

**Table 2** (continued)

| Author (date) | Country | Family-based Intervention and its description | Age range of children whose parents are included in the study and/or which the intervention targets | Participant Characteristics | Reports Mothers’ and/or Fathers’ perceptions separately:  Yes/No | Outcome Measures | Qualitative Data Collection Method | Qualitative Data Analysis Method | Robustness |
| --- | --- | --- | --- | --- | --- | --- | --- | --- | --- |
| Lee, Yelick, Brisebois and Banks  (2011) | USA | Family and Parenting programs  Description: This study discussed family and parenting programs more broadly in relation to improving parenting practices for child mental health and behavioural functioning. This study also acknowledged parenting programs as an intervention. | Not provided  Interventions target child mental health and behavioural functioning | *Total number of parents*: 17  *Sex of parents*: All male  *Age range of parents*: Not provided  *Ethnic group of parents*: Not provided  (Note: demographic data was only collected during the third focus group, and it is not specified how many participants attended the third focus group; thus, unable to accurately extract demographic data) | Yes  All participants are male/fathers  Assigned **fathers’ perceptions** | Fathers’ perceptions of barriers and facilitators to fathers’ engagement in family and parenting programs | Semi-structured focus groups | Content analysis | High |

**Table 2** (continued)

| Author (date) | Country | Family-based Intervention and its description | Age range of children whose parents are included in the study and/or which the intervention targets | Participant Characteristics | Reports Mothers’ and/or Fathers’ perceptions separately:  Yes/No | Outcome Measures | Qualitative Data Collection Method | Qualitative Data Analysis Method | Robustness |
| --- | --- | --- | --- | --- | --- | --- | --- | --- | --- |
| McPherson, Kerr, Casey and Marshall  (2017) | UK | Functional Family Therapy (FFT)  Description: Treating adolescent behavioural problems | 11-17 years | *Total number of parents*: 14 (all had completed the full FFT intervention)  *Sex of parents*: All female  *Age range of parents*: Not provided  *Ethnic group of parents*: Not provided | Yes  All participants are female/mothers  Assigned **mothers’ perceptions** | Barriers and facilitators to implementation and engagement of FFT | Semi-structured one-to-one interviews | Thematic analysis | High |

**Table 2** (continued)

| Author (date) | Country | Family-based Intervention and its description | Age range of children whose parents are included in the study and/or which the intervention targets | Participant Characteristics | Reports Mothers’ and/or Fathers’ perceptions separately:  Yes/No | Outcome Measures | Qualitative Data Collection Method | Qualitative Data Analysis Method | Robustness |
| --- | --- | --- | --- | --- | --- | --- | --- | --- | --- |
| Rahmqvist, Wells and Sarkadi  (2014) | Sweden | Triple P Parenting Program  Description: An evidence-based intervention targeting difficult child behaviour | Not provided; however, intervention defined as targeting children aged 0-16 years and targets child behavioural problems | *Total number of parents*: 10 (all had participated in Triple P)  *Sex of parents*: 7 females; 3 males  *Age range of parents*: 30 to 40 years  *Ethnic group of parents*: 5 (50%) Swedish mothers; 3 (30%) Swedish fathers; 1 (10%) Armenian mother; and 1 (10%) Syrian mother | Yes  Assigned **mothers’ and fathers’ separate perceptions** | Reasons for participating in Triple P; perceptions on the Triple P curriculum; and how the Triple P curriculum related to their parenting philosophy | Semi-structured interviews | Malterud’s method of Systematic Text Condensation | Moderate |

**Table 2** (continued)

| Author (date) | Country | Family-based Intervention and its description | Age range of children whose parents are included in the study and/or which the intervention targets | Participant Characteristics | Reports Mothers’ and/or Fathers’ perceptions separately:  Yes/No | Outcome Measures | Qualitative Data Collection Method | Qualitative Data Analysis Method | Robustness |
| --- | --- | --- | --- | --- | --- | --- | --- | --- | --- |
| Ruuskanen, Leitch, Sciberras and Evans  (2019) | Australia | Mindfulness Parenting Interventions (MPI)  Description: 8-week parenting training combining mindfulness strategies and behaviour management for ADHD symptomology for improving child behaviour | 7 to 11 years | *Total number of parents*: 13  *Sex of parents*: 11 females; 2 males  *Age range of parents*: only mothers’ ages could be provided, being 38 to 50 years, as the two fathers did not fill out the demographical information questionnaire  *Ethnic group of parents*: Not provided | No  84.6% of participants are female  Assigned **mothers’ perceptions** | Exploring parents’ barriers and facilitators to participation in MPI | Focus groups | Thematic analysis | High |

**Table 2** (continued)

| Author (date) | Country | Family-based Intervention and its description | Age range of children whose parents are included in the study and/or which the intervention targets | Participant Characteristics | Reports Mothers’ and/or Fathers’ perceptions separately:  Yes/No | Outcome Measures | Qualitative Data Collection Method | Qualitative Data Analysis Method | Robustness |
| --- | --- | --- | --- | --- | --- | --- | --- | --- | --- |
| Salinas, Smith and Armstrong (2011) | USA | Helping Our Toddlers, Developing Our Children’s Skills (HOT DOCS)  Description: 7-week behavioural parent training for managing and reducing challenging child behaviour | Not provided; however, intervention defined as for caregivers with toddlers and young children experiencing behaviour problems | *Total number of parents*: 13 (all had attended at least three HOT DOCS sessions)  *Sex of parents*: All male  *Age range of parents*: Except for 1 father who was <30 years (exact age not provided), remaining fathers were 30 to 49 years  *Ethnic group of parents*: 1 (7.7%) African American; 7 (53.8%) Hispanic; 5 (38.5%) White | Yes  All participants are male/fathers  Assigned **fathers’ perceptions** | Reasons for participation; useful strategies learned in treatment; barriers preventing participation; and suggestions for improving treatment for fathers | Focus groups | MAXQDA (2007 version); a qualitative data analysis software for identifying themes within transcriptions | High |

**Table 2** (continued)

| Author (date) | Country | Family-based Intervention and its description | Age range of children whose parents are included in the study and/or which the intervention targets | Participant Characteristics | Reports Mothers’ and/or Fathers’ perceptions separately:  Yes/No | Outcome Measures | Qualitative Data Collection Method | Qualitative Data Analysis Method | Robustness |
| --- | --- | --- | --- | --- | --- | --- | --- | --- | --- |
| Sicouri et al.  (2018) | Australia | Parenting interventions  Description: This study discussed parenting interventions more broadly including defining parenting interventions as treating child behavioural problems | 2-16 years | *Total number of parents*: 41  *Sex of parents*: All male  *Age range of parents*: Mean age of 41.78 years provided  *Ethnic group of parents*: Not provided | Yes  All participants are male/fathers  Assigned **fathers’ perceptions** | Barriers to engagement and preferences for parenting interventions | Focus groups | Inductive thematic analysis | High |

**Table 2** (continued)

| Author (date) | Country | Family-based Intervention and its description | Age range of children whose parents are included in the study and/or which the intervention targets | Participant Characteristics | Reports Mothers’ and/or Fathers’ perceptions separately:  Yes/No | Outcome Measures | Qualitative Data Collection Method | Qualitative Data Analysis Method | Robustness |
| --- | --- | --- | --- | --- | --- | --- | --- | --- | --- |
| Smith et al. (2015) | UK | Parenting Programmes (PPs) for preschool children with ADHD  Description: Early intervention approach for improving child outcomes and ADHD-related challenges | Not provided; however, PPs defined as treating ADHD-type problems in preschool children, including behavioural problems | *Total number of parents*: 25 (7 (28%) had no experience of PPs; 17 (68%) attended >1 group-based PP; and 1 (4%) attended an individual PP)  *Sex of parents*: All female  *Age range of parents*: 20 to 41+ years  *Ethnic group of parents*: 23 (92%) White; 2 (8%) Mixed race | Yes  All participants are female/mothers  Assigned **mothers’ perceptions** | Barriers to accessing and engaging in treatment suggestions for maximising ‘take-up’ and minimising ‘drop-out’; perceptions on how treatment could be improved to maximise effectiveness for families | Semi-structured interviews | Framework analysis | High |

**Table 2** (continued)

| Author (date) | Country | Family-based Intervention and its description | Age range of children whose parents are included in the study and/or which the intervention targets | Participant Characteristics | Reports Mothers’ and/or Fathers’ perceptions separately:  Yes/No | Outcome Measures | Qualitative Data Collection Method | Qualitative Data Analysis Method | Robustness |
| --- | --- | --- | --- | --- | --- | --- | --- | --- | --- |
| Stahlschmidt Threlfall, Seay, Lewis and Kohl  (2013) | USA | Triple P – Positive Parenting Program  Description:  Parenting intervention to strengthen parenting competencies and improve parent-child interactions, and recognises such programs as effective for treating child behaviour problems | 4 to 12 years | *Total number of parents*: 29  *Sex of parents*: All male  *Age range of parents*: Mean age of 37.45 years provided, and all parents were over 18 years of age  *Ethnic group of parents*: All African American | Yes  All participants are male/fathers  Assigned **fathers’ perceptions** | Perceptions about parenting programs and strategies to facilitate fathers’ engagement in parenting programs | Focus groups | Inductive approach utilising analytic triangulation, peer debriefing and support, and coding via NVivo (version 8) | High |

**Table 2** (continued)

| Author (date) | Country | Family-based Intervention and its description | Age range of children whose parents are included in the study and/or which the intervention targets | Participant Characteristics | Reports Mothers’ and/or Fathers’ perceptions separately:  Yes/No | Outcome Measures | Qualitative Data Collection Method | Qualitative Data Analysis Method | Robustness |
| --- | --- | --- | --- | --- | --- | --- | --- | --- | --- |
| Wilson, Weaver, Michelson and Day  (2018) | UK | The Helping Families Programme (HFP)  Description: 16-week community-based parenting intervention designed for parents with complex psychosocial needs, including Personality Disorder, to improve child mental health, child behaviour problems, and parent-child interactions | 3 to 11 years | *Total number of parents*: 5 (all had been referred to HFP)  *Sex of parents*: All female  *Age range of parents*: Not provided  *Ethnic group of parents*: Not provided | Yes  All participants are female/mothers  Assigned **mothers’ perceptions** | Experiences of help-seeking and participation in HFP (if applicable); and factors facilitating engagement in HFP | Semi-structured interviews | Interpretative Phenomenological Analysis (IPA) methods | High |

**Table 3 supplementary information.** Methodological quality assessment of included studies

| Authors (publication year) | Was there a clear statement of the aims of the research? | Is a qualitative methodology appropriate? | Was the research design appropirate to address the aims of the research? | Was the recruitment strategy appropriate to the aims of the research? | Was the data collected in a way that addressed the research issue? | Has the relationship between research and participants been adequately considered? | Have ethical issues been taken into consideration | Was the data analysis sufficiently rigorous? | Is there a clear statement of findings? | How valuable is the research? | Total score (max score = 10) |
| --- | --- | --- | --- | --- | --- | --- | --- | --- | --- | --- | --- |
| Attride-Stirling et al. (2014) | 1 (yes) | 1 (yes) | 1 (yes) | 1 (yes) | 1 (yes) | 0.5 (can’t tell^a^) | 1 (yes)* | 1 (yes) | 1 (yes) | 1 (yes) | 9.5 (high) |
| Coates et al. (2017) | 1 (yes) | 1 (yes) | 1 (yes) | 1 (yes) | 1 (yes) | 1 (yes) | 1 (yes) | 1 (yes) | 1 (yes) | 0.5 (can’t tell) | 9.5 (high) |
| Dolan (2014) | 1 (yes) | 1 (yes) | 1 (yes) | 1 (yes) | 1 (yes) | 1 (yes) | 1 (yes) | 1 (yes) | 1 (yes) | 0.5 (can’t tell) | 9.5 (high) |
| Dorsey et al. (2014) | 1 (yes) | 1 (yes) | 1 (yes) | 1 (yes) | 1 (yes) | 0.5 (can’t tell) | 1 (yes) | 1 (yes) | 1 (yes) | 1 (yes) | 9.5 (high) |
| Frank et al. (2015) | 1 (yes) | 1 (yes) | 1 (yes) | 1 (yes) | 1 (yes) | 0.5 (can’t tell) | 1 (yes) | 1 (yes) | 1 (yes) | 1 (yes) | 9.5 (high) |
| Friars and Mellor (2009) | 1 (yes) | 1 (yes) | 1 (yes) | 1 (yes) | 0.5 (can’t tell) | 0 (no) | 1 (yes) | 0 (no) | 1 (yes) | 1 (yes) | 7.5 (moderate) |
| Furlong and McGilloway (2012) | 1 (yes) | 1 (yes) | 1 (yes) | 1 (yes) | 1 (yes) | 1 (yes) | 0 (no)* | 1 (yes) | 1 (yes) | 1 (yes) | 9 (high) |
| Fox et al. (2017) | 1 (yes) | 1 (yes) | 1 (yes) | 0.5 (can’t tell) | 1 (yes) | 0 (no) | 0 (no)* | 1 (yes) | 1 (yes) | 1 (yes) | 7.5  (moderate) |
| Gopalan et al. (2015) | 1 (yes) | 1 (yes) | 1 (yes) | 1 (yes) | 1 (yes) | 0.5 (can’t tell) | 1 (yes) | 1 (yes) | 1 (yes) | 1 (yes) | 9.5 (high) |
| Huntington and Vetere (2015) | 1 (yes) | 1 (yes) | 1 (yes) | 0 (no) | 1 (yes) | 0.5 (can’t tell) | 0 (no)* | 1 (yes) | 1 (yes) | 1 (yes) | 7.5 (moderate) |
| Kerwin et al. (2014) | 1 (yes) | 1 (yes) | 1 (yes) | 1 (yes) | 1 (yes) | 0.5 (can’t tell) | 1 (yes) | 0.5 (can’t tell) | 1 (yes) | 0.5 (can’t tell) | 8.5 (moderate) |
| Lee et al. (2011) | 1 (yes) | 1 (yes) | 1 (yes) | 1 (yes) | 1 (yes) | 0.5 (can’t tell) | 1 (yes) | 0.5 (can’t tell) | 1 (yes) | 1 (yes) | 9 (high) |
| McPherson et al. (2017) | 1 (yes) | 1 (yes) | 1 (yes) | 1 (yes) | 1 (yes) | 1 (yes) | 1 (yes) | 1 (yes) | 1 (yes) | 1 (yes) | 10 (high) |
| Authors (publication year) | Was there a clear statement of the aims of the research? | Is a qualitative methodology appropriate? | Was the research design appropirate to address the aims of the research? | Was the recruitment strategy appropriate to the aims of the research? | Was the data collected in a way that addressed the research issue? | Has the relationship between research and participants been adequately considered? | Have ethical issues been taken into consideration | Was the data analysis sufficiently rigorous? | Is there a clear statement of findings? | How valuable is the research? | Total score (max score = 10) |
| Rahmqvist et al. (2014) | 1 (yes) | 1 (yes) | 1 (yes) | 1 (yes) | 1 (yes) | 0 (no) | 0.5 (can’t tell) | 1 (yes) | 1 (yes) | 1 (yes) | 8.5 (moderate) |
| Ruuskanen et al (2019) | 1 (yes) | 1 (yes) | 1 (yes) | 1 (yes) | 1 (yes) | 0.5 (can’t tell) | 1 (yes) | 1 (yes) | 1 (yes) | 1 (yes) | 9.5 (high) |
| Salinas et al. (2011) | 1 (yes) | 1 (yes) | 1 (yes) | 1 (yes) | 1 (yes) | 1 (yes) | 1 (yes)* | 1 (yes) | 1 (yes) | 0.5 (can’t tell) | 9.5 (high) |
| Sicouri et al. (2018) | 1 (yes) | 1 (yes) | 1 (yes) | 1 (yes) | 1 (yes) | 0.5 (can’t tell) | 1 (yes) | 1 (yes) | 1 (yes) | 1 (yes) | 9.5 (high) |
| Smith et al. (2015) | 1 (yes) | 1 (yes) | 1 (yes) | 1 (yes) | 1 (yes) | 0.5 (can’t tell) | 1 (yes) | 1 (yes) | 1 (yes) | 1 (yes) | 9.5 (high) |
| Stahlschmidt et al. (2013) | 1 (yes) | 1 (yes) | 1 (yes) | 1 (yes) | 1 (yes) | 1 (yes) | 1 (yes) | 1 (yes) | 1 (yes) | 1 (yes) | 10 (high) |
| Wilson et al. (2018) | 1 (yes) | 1 (yes) | 1 (yes) | 1 (yes) | 1 (yes) | 0.5 (can’t tell) | 0.5 (can’t tell) | 1 (yes) | 1 (yes) | 1 (yes) | 9 (high) |

^a^ ‘can’t tell’ indicates that the required information was insufficient or unclear

* indicates no report of ethical approval

**Table 4 supplementary information.** Mothers’ and fathers’ reported barriers to engaging in family-based interventions. Letters in brackets represent each theme’s corresponding study.

| **Mothers’ Barriers** | **Fathers’ Barriers** |
| --- | --- |
| **1. Psychological**   - **Stigma (^r, t^)**   *Judged as a ‘bad parent’; shame/embarrassment (e.g., of family problems)*   - **Distrust (^d, g, i, r^)**   *Confidentiality concerns; distrust of practitioner’s affiliated system/organisation; social services investigations resulting from disclosures*   - **Parental mental health (^f, i, o^)**   *Depression; ADHD; stress*   - **Attitudes/Beliefs (^a, f, r^)**   *Considering the intervention as non-beneficial; problem is within the child and not parenting; not ready/motivated/able to make changes to own behaviour; missing a session means you cannot continue (e.g., because future content relates back to a missed session)* | **1. Psychological**   - **Stigma (^c, l^)**   *Judged as a ‘bad parent’; shame/embarrassment (e.g., of family problems); help-seeking considered as weak/not coping; fear of ridicule for attending; associating family-based interventions with Child Protection Services*   - **Distrust (^n^)**   *Confidentiality concerns* |
| **2. Situational factors**   - **Competing demands (^a, d, f, g, k, n, o, r^)**   *Work commitments; busy schedule; childcare responsibilities; illness; caring for sick relatives; housework*   - **Practical (^d, f, i, k, m, o, r^)**   *Transport difficulties; inconvenient timings; long waiting times for enrolment and the programme starting; financial difficulties with affording treatment (particularly amongst single mothers); lengthy homework tasks; child refusing to attend (i.e., multicomponent treatments)*   - **Demographic (f^, g, r^)**   *Single parenting; young parent; living in a disadvantaged community; having several children* | **2. Situational factors**   - **Competing demands (^e, n, p, q, s^)**   *Work commitments; busy schedule; childcare responsibilities*   - **Practical (^q, s^)**   *Transport difficulties; inconvenient timings* |
| **3.** **Lack of knowledge/awareness (^a, r^)**  *Lack of knowledge/awareness of existing family-based interventions; unclear objectives and expectations of treatment* | **3. Lack of knowledge/awareness (^q, s^)**  *Lack of knowledge/awareness of existing family-based interventions; unclear objectives and expectations of treatment* |
| **4. Programme/Intervention experiences**   - **Content (^b, f, g, i, o, r^)**   *Overly clinical or spiritual language; not tailored to parents’/child’s needs; not aligned to parents’ culture; strategies cannot be applied to more than one child*   - **Poor Orientation (^d^)**   *Poor orientation to practitioner/agency*   - **Negative past experiences (^d, g^)**   *Negative past experiences of mental health services; parents’ own negative parenting history being triggered*   - **Perceptions of inadequate treatment (^d, r^)**   *Perceived lack of family-based interventions for foster carers; expected improvements not occurring quickly increased dropout* | **4. Programme/Intervention experiences**   - **Content (^e, n, q^)**   *Overly academic/lecture-based language; not tailored to parents’/child’s needs; mother-focused; ‘too’ basic*   - **Perceptions of family-based interventions (^c, l, q, s^)**   *Assumptions of ‘legitimate’ users (e.g., mothers, problematic fathers, rich parents, parents whose children have serious behaviour problems); fewer opportunities for fathers to participate compared to mothers* |
| **5. Co-parenting (^g, k^)**  *Unsupportive co-parents; parenting conflict (both of which obstructed the implementation of learned strategies)* | **5. Co-parenting (^p, q^)**  *Unsupportive co-parents; parenting conflict (both of which obstructed the implementation of learned strategies)* |
| **6. Group therapy experiences**   - **Group differences (^f, g, i, r^)** *Family demographics (e.g., age/education/income/marital status/socio-cultural); single parents not feeling like they ‘fit in’ with co-parents; individual differences in child symptom severity* - **Fear/Worries (^d, f, g, r^)** *Not ‘fitting’ in with the group; uncomfortable talking in front of a group; slipping into ‘old’ parenting styles without group support; low self-confidence in attending groups alone; overwhelmed by quantity of required parental involvement*   **7. Practitioner characteristics (^d, f, i^)**  *Unable to manage group (e.g., poor time-management, not allowing everyone the chance to speak); poor parent-practitioner interactional style (e.g., interactional style does not ‘fit’ with parents’ language/culture); inadequate understanding of child problems/inexperienced* | **6. Father involvement**   - **Beliefs of fatherhood/masculinity (^c, q^)**   *Considering fathers as not primary caregivers; active involvement in family-based interventions conflicts with traditional father/male ‘provider’ role; difficulties expressing emotion*   - **Maternal gatekeeping (^e, q^)**   *Controlling in-session conversation; discouraging father participation; unwilling to share with mother present* |

^a^Attride-Stirling et al. (2004)

^b^ Coates et al. (2017)

^c^ Dolan (2014)

^d^ Dorsey et al. (2014)

^e^ Frank et al. (2015)

^f^ Friars and Mellor (2009)

^g^ Furlong and McGilloway (2012)

^h^ Fox et al. (2017)

^i^ Gopalan et al. (2015)

^j^ Huntington and Vetere (2015)

^k^ Kerwin et al. (2014)

^l^ Lee et al. (2011)

^m^ McPherson et al. (2017)

^n^ Rahmqvist et al. (2014)

^o^ Ruuskanen et al. (2019)

^p^ Salinas et al. (2011)

^q^ Sicouri et al. (2018)

^r^ Smith et al. (2015)

^s^ Stahlschmidt et al. (2013)

^t^ Wilson et al. (2018)

**Table 5 supplementary information.** Mothers’ and fathers’ reported facilitators to engaging in family-based interventions. Letters in brackets represent each theme’s corresponding study.

| **Mothers’ Facilitators** | **Fathers’ Facilitators** |
| --- | --- |
| **1. Practitioner characteristics (^a, g, i, j, m, n, o, r, t^)**  *Non-judgemental; inclusive of both parents; qualified/experienced/knowledgeable; manages group well; is a parent themselves; empowers (rather than dictates) parenting decisions; enquires about parent’s preferred interactional style* | **1. Practitioner characteristics (^e, p, q^)**  *Non-judgemental; inclusive of both parents; qualified/experienced/knowledgeable; male practitioner for father-only groups; aware of child’s needs at outset; observes child/ father-child interactions* |
| **2. Group therapy experiences (^b, g, i, n, o, r^)** *Peer support; learning from others; group homogeneity (e.g., grouping parents with similar needs/experiences/struggles); egalitarian and non-judgemental relationships between parents within a group; trust/confidentiality* | **2. Group therapy experiences (^c, e, p, q^)** *Peer support; learning from others; group homogeneity (e.g., grouping parents with similar needs/experiences/struggles); trust/confidentiality* |
| **3. Situational factors**   - **Convenient location (^a, d, l, m, o^)**   *Local/community settings (e.g., school, community centres); home; same location; non-threatening venue (e.g., bright learning space)*   - **Convenient timings (^a, d, f, n, o^)** *Flexible/varying timings; shorter duration of sessions; reduced number of sessions; weekday evenings* | **3. Situational factors**   - **Convenient location** **(^e, p, q^)** *Local/community settings (e.g., school, community centres); varying location; leaving home; parking/transport access* - **Convenient timings** **(^p, q^)** *Flexible/varying timings; after-work hours; weekends; shorter duration but increased number of sessions overall (e.g., to practice parenting strategies or have follow-ups)* |
| **4. Programme/Intervention experiences**   - **Content (^b, d, g, m, n, r, t^)**   *Tailored to parents’/child’s needs; structured; fun homework tasks; practical strategies*   - **Delivery (^f, i, o, r^)**   *Accessible/early provision of resources; resources in varying formats; comfortable environment; activity-based; one-to-one work*   - **Incentives (^i^)**   *Travel expenses; refreshments; rewards/reinforcements for engagement*   - **Additional support (^b, g, o, r^)**   *Optional counselling for parents’ own mental health; refresher course; home visits; follow-ups; telephone session reminders; online forums (e.g., to support those parents who cannot attend face-to-face)* | **4. Programme/Intervention experiences**   - **Content (^c, e, p, q^)** *Tailored to parents’/child’s needs; self-care; managing and normalising emotions (e.g., learning how to show physical affection); problem-solving challenging child behaviour; decision-making strategies; effective co-parenting; fathers’ role in child development* - **Delivery (^c, e, q^)** *Accessible resources; comfortable environment; activity-based; in-person sessions combined with online resources; father-only groups; discussion groups* - **Incentives (^e, p, q, s^)** *Travel expenses; refreshments; vouchers/gift-cards; certificate of completion; childcare provision* - **Additional support (^q^)** *Follow-ups (e.g., to troubleshoot specific issues)* |
| **5. Co-parenting (^g, i,^)**  *Provisions for the participation of both mothers and fathers in a parenting team; bonding/teamwork* | **5. Co-parenting (^e, p, q^)**  *Provisions for the participation of both mothers and fathers in a parenting team; mothers encouraging father participation* |
| **6. Knowledge/Awareness**   - **Evidence-based intervention (^a, d, i, m, n, o, r^)** *Acknowledging that the intervention is supported by empirical evidence* - **Perceived intervention benefits** **(^a, d, i, m, n, o, r^)** *Perceiving the intervention to help; acknowledging improvements from participation* | **6. Knowledge/Awareness**   - **Evidence-based intervention (^p^)** *Acknowledging that the intervention is supported by empirical evidence* - **Perceived intervention benefits** **(^p^)** *Perceiving the intervention to help; acknowledging improvements from participation* |
| **7. Explicit Engagement Stages**   - **Help-seeking/Enrolment (^a, i, m, n, o, t^)**   *Feeling overwhelmed/helpless/desperate (particularly regarding improving child behaviour); family ‘crisis point’; recognised need for support; endeavouring to avoid child-welfare involvement; anticipating treatment benefits from attending*   - **Treatment attendance/retention (^a, h, i, m, o^)**   *Rewards/reinforcements; incentives; motivation to complete treatment; improved co-parenting; perceiving that retention will aid parents’ mental illness; small attendance fee; experiencing/witnessing treatment benefits; culturally informed practice*   - **Treatment in-session participation (^i^)**   *Improvements in co-parenting (e.g., co-parents bonding/connecting)*   - **Enactment (^o, r^)**   *Refresher courses/check-ins/follow-ups; co-parent/father/family support; incorporating learned strategies into daily routine* | **7. Explicit Engagement Stages**   - **Help-seeking/Enrolment (^c, p, s^)** *Seeking to improve as a parent* - **Treatment attendance/retention (^c, p, s^)** *Seeking to improve as a parent* |
| **8. Advertisement/Recruitment**   - **Various advertising formats (^r^)** *Information at their General Practice (i.e., community healthcare service); leaflets; word-of-mouth; hearing experiences from a previous attendee* - **Messages (^d, i, r^)**  *Orientation to the intervention and parental participation requirements* | **8. Advertisement/Recruitment**   - **Various advertising formats (^e, q, s^)**   *leaflets; word-of-mouth; hearing experiences from a previous attendee; billboards; TV/radio/newspapers; endorsements by mothers, credible figures, or organisations*   - **Messages (^e, q^)**   *Father-friendly messages (e.g., do not imply that fathers are doing a bad job); father-relatable material (e.g., images of fathers from different races/ethnicities); use of humour; orientation to the intervention and parental participation requirements* |
|  | **9. Father involvement (^c^)**  *Accepting the ideology/belief of an actively and emotionally engaged father* |

^a^Attride-Stirling et al. (2004)

^b^ Coates et al. (2017)

^c^ Dolan (2014)

^d^ Dorsey et al. (2014)

^e^ Frank et al. (2015)

^f^ Friars and Mellor (2009)

^g^ Furlong and McGilloway (2012)

^h^ Fox et al. (2017)

^i^ Gopalan et al. (2015)

^j^ Huntington and Vetere (2015)

^k^ Kerwin et al. (2014)

^l^ Lee et al. (2011)

^m^ McPherson et al. (2017)

^n^ Rahmqvist et al. (2014)

^o^ Ruuskanen et al. (2019)

^p^ Salinas et al. (2011)

^q^ Sicouri et al. (2018)

^r^ Smith et al. (2015)

^s^ Stahlschmidt et al. (2013)

^t^ Wilson et al. (2018)
